# Which population level environmental factors are associated with asthma, rhinoconjunctivitis and eczema? Review of the ecological analyses of ISAAC Phase One

**DOI:** 10.1186/1465-9921-11-8

**Published:** 2010-01-21

**Authors:** M Innes Asher, Alistair W Stewart, Javier Mallol, Stephen Montefort, Christopher KW Lai, Nadia Aït-Khaled, Joseph Odhiambo, The ISAAC Phase One Study Group

**Affiliations:** 1 Department of Paediatrics: Child and Youth Health, The University of Auckland, Auckland, New Zealand; 2 School of Population Health, The University of Auckland, Auckland, New Zealand; 3 Department of Pediatric Respiratory Medicine, University of Santiago de Chile (USACH), Hospital El Pino, Santiago, Chile; 4 Department of Medicine, University of Malta, Malta; 5 Department of Medicine and Therapeutics, The Chinese University of Hong Kong, Hong Kong, SAR PR China; 6 International Union Against Tuberculosis Lung Diseases, Paris, France; 7 Centre for Respiratory Diseases Research, Kenya Medical Research Institute (KEMRI), Nairobi, Kenya; 8 Members listed at the end of the manuscript

## Abstract

The International Study of Asthma and Allergies in Childhood (ISAAC) Phase One showed large worldwide variations in the prevalence of symptoms of asthma, rhinoconjunctivitis and eczema, up to 10 to 20 fold between countries. Ecological analyses were undertaken with ISAAC Phase One data to explore factors that may have contributed to these variations, and are summarised and reviewed here.

In ISAAC Phase One the prevalence of symptoms in the past 12 months of asthma, rhinoconjunctivitis and eczema were estimated from studies in 463,801 children aged 13 - 14 years in 155 centres in 56 countries, and in 257,800 children aged 6-7 years in 91 centres in 38 countries. Ecological analyses were undertaken between symptom prevalence and the following: Gross National Product per capita (GNP), food intake, immunisation rates, tuberculosis notifications, climatic factors, tobacco consumption, pollen, antibiotic sales, paracetamol sales, and outdoor air pollution.

Symptom prevalence of all three conditions was positively associated with GNP, trans fatty acids, paracetamol, and women smoking, and inversely associated with food of plant origin, pollen, immunisations, tuberculosis notifications, air pollution, and men smoking. The magnitude of these associations was small, but consistent in direction between conditions. There were mixed associations of climate and antibiotic sales with symptom prevalence.

The potential causality of these associations warrant further investigation. Factors which prevent the development of these conditions, or where there is an absence of a positive correlation at a population level may be as important from the policy viewpoint as a focus on the positive risk factors. Interventions based on small associations may have the potential for a large public health benefit.

## What was ISAAC Phase One?

The International Study of Asthma and Allergies in Childhood (ISAAC) Phase One studied symptom prevalence and severity of asthma, rhinoconjunctivitis and eczema between populations around the world to elucidate factors influencing these conditions. This multi-centre cross-sectional study was undertaken between 1992 and 1997 using standardised methodology with subjects being school children from schools which were randomly sampled within centres [1]. Two age groups of children (13-14 and 6-7 years) were studied, with a target sample size of 3,000 children per age group per centre. Simple written questionnaires were self-completed by the 13-14 year old participants, and by the parents of the 6-7 year old participants. These questionnaires were translated from English into a local language where appropriate. An optional video asthma questionnaire was delivered to the 13-14 year age group, with verbal instructions in the local language. The participation rates averaged 92% for the 13-14 year age group and 89% for the 6-7 year age group. The findings of large variations in the symptom prevalence of asthma, rhinoconjunctivitis and eczema, up to 10 to 20 fold between countries, around the world [2-5] suggested that environmental rather than genetic factors may be most influential.

One of the scientific driving forces behind the ISAAC collaboration was the suspicion that causes of asthma in high prevalence countries might be so widespread that it would be difficult if not impossible to detect them by comparisons between individuals within those countries. The very large number of participating centres (156) and countries (56) in ISAAC Phase One was an ideal opportunity to look at potential causes between populations. Ecological analyses are an appropriate method to do this to generate hypotheses which may explain these variations. Thus ecological analyses were undertaken with ISAAC Phase One symptom prevalence estimates, using a wide range of data sources at a population level, on a variety of environmental topics [6-17]. The findings for the potentially protective or risk factors examined which are relevant for asthma, rhinoconjunctivitis and eczema are reviewed and interpreted in this paper.

## What ecological analyses were undertaken?

Ecological analyses were undertaken with ISAAC Phase One symptom prevalence data for asthma, rhinoconjunctivitis and eczema with environmental data from existing sources. In some cases environmental data was unavailable for all centres or countries. The effect of country level economic development was examined using the 1993 Gross National Product per capita (GNP) as reported by the World Bank for all 156 centres in 56 countries involved in Phase One [14]. Dietary factors were assessed using Food and Agriculture Organization FAOSTAT food balance sheets for 1995 in 53 countries. Food produced and imported, less food exported gave the country supply of food from which was estimated the per capita supply of food [9]. Associations with consumption of *trans *fatty acids were assessed in 55 centres in 10 European countries using data from 1999 [17]. Immunisation rates at a national level were obtained from the World Health Organization (WHO) for 56 countries, and immunisation rates at a centre level were obtained through ISAAC Principal Investigators in 92 ISAAC centres for vaccines against diphtheria, tetanus, pertussis, BCG and measles [7]. The years selected for the immunisation data (1982 for the 13-14 year age group and 1989 for the 6-7 year age group) approximately corresponded to the year of birth of the participants. Tuberculosis (TB) exposure was assessed in two ways - firstly using notification rates from WHO data for 1980-1982 in 23 countries for the 13-14 year age group at the time when the older age group would have been infants [15]; secondly from WHO TB estimated incidence for the 6-7 year age group in 38 countries and 55 countries for the 13-14 year age group in 1995, the mean year of the data collection for the older age group [13]. Climatic factors were assessed using data from the World Weather Guide and derived variables for 146 ISAAC centres [16] and a subset of 57 centres in 12 countries in Western Europe. Tobacco exposure was assessed using WHO data for tobacco consumption per capita 1990-1992, and tobacco smoking prevalence in women and tobacco smoking in men in 155 centres from 56 countries [11]. Pollen exposure was assessed using data for pollen counts, and duration and severity of pollen seasons in 28 centres in 11 countries for 1995 [8]. Associations with antibiotic use was assessed using data for antibiotic sales for 28 countries from International Medical Statistics, Health Global Services, United Kingdom 1995 [10]. Air pollution was assessed for 105 centres in 51 countries using the World Bank Global Model on Ambient Particulates for 1999 which estimated annual concentrations for all cities with populations of more than 100,000 [6]. Paracetamol exposure was assessed using data for paracetamol sales per capita in 106 centres [12].

Each analysis is described in detail elsewhere [6-17]. For all analyses mixed model regression analyses were carried out to incorporate the country and centre structure of the data sets, except *trans *fatty acids where linear regressions were used. ISAAC data was at the centre level, but most environmental data sets represented whole countries, therefore mostly country level was used in the analyses as specified in the source articles. Exceptions were air pollution and climate which could be analysed at centre (city) level. A variety of measures of association were used for the analyses such as Spearman rank correlation coefficient, and the statistical significance level used was p < 0.05. Non-statistically significant trends were noted where they were consistent with significant findings. The analyses did not examine combinations and interactions of the variables.

As GNP was found to have a weak positive association with symptom prevalence [14], the analyses of other environmental factors were repeated with GNP in the model. The findings presented in this paper pertain to analyses adjusted for GNP, but not adjusted for any other variables. Although the published papers analysed most of the ISAAC questions, this paper reviews only the analyses of symptoms in the past 12 months of asthma, rhinoconjunctivitis and eczema.

## What were the key findings?

The ecological analyses in both age groups demonstrated mostly weak relationships of small magnitude between environmental variables and current symptoms of asthma, rhinoconjunctivitis and eczema, or no association at all. The results are summarised in Table 1, Table 2 and Table 3. Where associations were found these were generally in the same direction for all three conditions, but were statistically significant for only some associations [6-9,11,12,14,17]. A weak positive association was found between asthma and eczema symptoms and GNP. Weak positive associations were found between symptom prevalence and tobacco consumption, smoking by women, *trans *fatty acids and paracetamol. Weak inverse associations were found for plant-based foods, immunisation for DTP and measles, tuberculosis incidence pollen exposure, smoking by men. A mixed pattern of associations were found for antibiotic sales and climate. For air pollution, in those countries with more than one centre, the association was weakly positive, in contrast to the weakly negative association at country level.

**Table 1:** Symptoms of wheeze in the past 12 months - summary of ecological analyses*.

Ecological Issue	Specific Factor	13-14 Year Age Group				6-7 Year Age Group	
		
		Written Questionnaire		Video Questionnaire		Written Questionnaire	
		
		Direction of effect	P Value	Direction of effect	P Value	Direction of effect	P value
Economic development	GNP	+	0.007	+	0.08	+	0.84

Diet	Calories from cereals and rice %	-	0.0005	-	0.07	-	0.021
	Protein from cereals and nuts %	-	0.002	-	0.015	-	0.062
	Starch g/day	-	0.008	-	0.01	-	0.036
	Vegetables g/d	-	0.041	-	0.205	-	0.262
	Vegetable Vitamin A μ/d	-	0.04	-	0.159	-	0.235
	*Trans *fatty acids	+	< 0.0001	+	< 0.05		

Immunisation	Local immunisation rates for DTP#	-	0.047			-	> 0.05
	Local immunisation rates for measles	-	> 0.05			-	> 0.05

TB exposure	TB notification rates	-	0.344	-	0.018		
	Estimated TB incidence rates	-	0.262	-	0.263	-	< 0.0001

Outdoor climate	Altitude	-	> 0.05	+	> 0.05	-	> 0.05
	Annual variation in mean temperature	-	> 0.05	-	> 0.05	-	> 0.05
	Lowest monthly mean temperature	+	> 0.05	**0**	> 0.05	+	> 0.05
	Annual variation in mean relative humidity	-	> 0.05	+	> 0.05	-	> 0.05
	Lowest monthly mean relative humidity	**0**	> 0.05	-	> 0.05	**0**	< 0.05
	Mean relative humidity of ≥ 1 month < 50%humidity	+	> 0.05	+	> 0.05	+	> 0.05

Indoor climate	Mean annual relative humidity	**0**	> 0.05	-	> 0.05	-	> 0.05
	Lowest monthly mean relative humidity	+	> 0.05	**0**	> 0.05	**0**	> 0.05

Tobacco smoking	Tobacco consumption, ≥ 15 yrs	**0**	0.92	+	0.53	+	0.46
	Tobacco smoking, women	+	0.06	+	0.72	+	0.32
	Tobacco smoking, men	-	0.001	-	0.004	-	0.06

Pollen	Total grass pollen count	-	0.67				
	Days with high pollen counts	-	0.39				

Antibiotic usage	Antibiotic sales per capita	-	0.21	+	0.01		

Air pollution	PM_10_	-	0.03			-	0.06

Paracetamol	Per capita paracetamol sales	+	< 0.0005			+	0.001

*standardized for GNP. Where p values are recorded as > 0.05 the non significant p values were not provided in the source publication. #DTP = Diphtheria and tetanus toxoids and pertussis.

Blank space = data not collected. + = positive association. - = inverse association. 0 = no association.

**Table 2:** Symptoms of rhinoconjunctivitis in the past 12 months - summary of ecological analyses*.

Ecological Issue	Specific Factor	13-14 Year Age Group		6-7 Year Age Group	
		
		Direction of effect	P Value	Direction of effect	P Value
Economic development	GNP	+	0.12	0.06	0.32

Diet	Calories from cereals and rice %	-	0.007	-	0.083
	Protein from cereals and nuts %	-	0.039	-	0.111
	Starch g/d	-	0.266	-	0.069
	Vegetables g/d	-	0.083	-	0.453
	Vegetable Vitamin A μ/d	-	0.085	-	0.479
	*Trans *fatty acids	+	< 0.0001		

Immunisation	Local immunisation rates for DTP#	+	0.002	-	> 0.05
	Local immunisation rates for measles	-	0.015	-	> 0.05

TB exposure	TB notification rates, WHO	-	0.015		

Outdoor climate	Altitude	-	> 0.05	-	> 0.05
	Annual variation in mean temperature	-	> 0.05	-	> 0.05
	Lowest monthly mean temperature	+	> 0.05	+	> 0.05
	Annual variation in mean relative humidity	+	> 0.05	-	> 0.05
	Lowest monthly mean relative humidity	-	> 0.05	-	> 0.05
	Mean relative humidity of ≥ 1 month < 50%humidity	+	> 0.05	+	> 0.05

Indoor climate	Mean annual relative humidity	+	> 0.05	+	> 0.05
	Lowest monthly mean relative humidity	+	> 0.05	+	> 0.05

Tobacco smoking	Tobacco consumption, ≥ 15 yrs	-	0.86	+	0.52
	Tobacco smoking, women	-	0.68	+	0.76
	Tobacco smoking, men	-	< 0.001	-	0.06

Pollen	Total grass pollen count	-	0.07		
	Days with high pollen counts	-	0.14		

Antibiotic usage	Antibiotic sales per capita	-	0.76		

Air pollution	PM_10_	-	0.49	-	0.06
Paracetamol	Per capita paracetamol sales	+	< 0.0005	+	0.002

*standardized for GNP. Where p values are recorded as > 0.05 the non significant p values were not provided in the source publication. #DTP = Diphtheria and tetanus toxoids and pertussis.

Blank space = data not collected. + = positive association. - = inverse association.

**Table 3:** Symptoms of eczema in the past 12 months - summary of ecological analyses*.

Ecological Issue	Specific Factor	13-14 Year Age Group		6-7 Year Age Group	
		
		Direction of effect	P Value	Direction of effect	P Value
Economic development	GNP	+	0.05	+	0.02

Diet	Calories from cereals and rice %	-	0.246	-	0.767
	Protein from cereals and nuts %	-	0.028	-	0.07
	Starch g/d	-	0.560	-	0.058
	Vegetables g/d	-	0.001	-	0.157
	Vegetable Vitamin A μ/d	-	0.001	-	0.153
	*Trans *fatty acids	+	< 0.0001		

Immunisation	Local immunisation rates for DTP#	-	0.048	-	> 0.05
	Local immunisation rates for measles	-	0.036	-	> 0.05

TB exposure	TB notification rates, WHO	+	0.544		

Outdoor climate	Latitude	**0**	> 0.05	+	> 0.05
	Altitude	+	> 0.05	+	> 0.05
	Mean annual temperature	-	> 0.05	-	> 0.05
	Annual variation in mean temperature	+	< 0.05	+	> 0.05
	Lowest monthly mean temperature	-	> 0.05	-	< 0.05
	Annual variation in mean relative humidity	-	> 0.05	-	> 0.05
	Lowest monthly mean relative humidity	-	> 0.05	-	> 0.05

Indoor climate	Mean annual relative humidity	-	> 0.05	-	< 0.05
	Lowest monthly mean relative humidity	**0**	> 0.05	-	> 0.05

Tobacco smoking	Tobacco consumption, ≥ 15 yrs	+	0.35	+	0.87
	Tobacco smoking, women	+	0.42	+	0.17
	Tobacco smoking, men	-	0.007	-	0.67

Pollen	Total grass pollen count	-	0.15		
	Days with high pollen counts	-	0.32		

Antibiotic usage	Antibiotic sales per capita	-	0.46		

Air pollution	PM_10_	-	0.07	-	0.10

Paracetamol	Per capita paracetamol sales	+	< 0.0005	+	0.001

*standardized for GNP. Where p values are recorded as > 0.05 the non significant p values were not provided in the source publication. #DTP = Diphtheria and tetanus toxoids and pertussis.

Blank space = data not collected. + = positive association. - = inverse association. 0 = no association.

## What do these findings mean? - methodological issues

This is the first large worldwide analysis of protective and risk factors related to asthma, rhinoconjunctivitis and eczema in children between populations. Standardised methodology was used to estimate the 12 month period prevalence of symptoms of the three conditions. The recall of 12 month symptoms is a more accurate assessment of disease than diagnostic labelling which is subject to recall bias and different diagnostic fashions especially in such diverse parts of the world.

As country level environmental data was used most commonly (because centre-level data was unavailable) associations at the country level may have introduced complex biases when analysed against centre-level symptoms, and thus some caution is required in the interpretation of the results. This is illustrated by the contrasting results for air pollution.

The global coverage of ISAAC is advantageous in terms of scope and size for ecological analyses, making them potentially more robust than smaller less representative studies. Multiple languages were used which raises the possibility of population level confounding. However the variations in prevalence of asthma from the written questionnaire were similar in their global distribution to the prevalence from the video questionnaire [2]. Furthermore recent analysis suggests that translations of the written questionnaire are reasonably accurate [18], and several of the papers have reported consistent findings for within-region or within-language comparisons which is reassuring. The most robust comparisons, from this viewpoint, are those based on comparisons between centre-level symptom prevalence values and centre level environmental exposures as occurred for the air pollution [6] and immunisation [7] analyses. Further, the results of parental responses for the younger children were similar to those of the responses from the older children.

The ecological approach attempted to find factors which explained the large international variation, but we are unable to tell which factors are relevant to asthma causation. For example if everyone smoked then smoking wouldn't explain any of the international variation, even if it was an important risk factor for asthma (although if there was a strong dose response an effect might be found). It is not appropriate to compare the size of the correlation coefficients, as the methods for calculating these distort the effects, so that the magnitude of effect cannot be interpreted from them [19]. Notwithstanding these caveats, there are plausible mechanisms for each association (discussed below), suggesting that various factors associated with human development may be affecting variations in symptom prevalence between populations.

Explaining patterns of diseases across the world one variable at a time may be fraught with difficulties, as different factors are likely to be more important in different regions of the world. For example, climate may be important in countries where there is variation in temperature and humidity, and TB exposure will be of potential importance where TB occurs, and not where it doesn't occur. It is also likely that these diseases are multi component diseases, i.e. 3 or more factors need to be present before disease may become manifest, but that level of analysis has not been undertaken.

### Economic factors

The associations observed suggest that economic development of a country may bring with it changes influencing asthma, rhinoconjunctivitis and eczema [14]. Further supportive evidence of this association was found recently in ISAAC Phase Three where data from 98 countries showed that the prevalence of asthma symptoms showed a positive relationship with Gross National Income (GNI), although the prevalence of severe symptoms correlated inversely with GNI [20]. However caution should be used in interpreting the findings because of the great inequalities in income distribution within almost all countries in developing regions of the world. GNP represents the total economic activity of the country, reflecting mean wealth rather than median wealth. Thus countries with a highly skewed income distribution due to concentration of wealth in the hands of a small fraction of the population may have a relatively high per capita GNP while the majority of citizens have a relatively low level of income [21]. A further consideration is that GNP does not measure factors that affect quality of life, such as the quality of the environment.

### Diet

Dietary patterns have changed rapidly with modernisation or westernisation, and the associated move away from plant-based foods and addition of man-made fats might affect symptom prevalence. No associations were found for meat, and milk, but showed a pattern of inverse association between plant-based food and symptoms of the three conditions [9]. A protective effect of plant-based food might be mediated through effects on intestinal microflora which are necessary for maturation of Th1 immunity [22]. A further mechanism may be mediated through antioxidant content. This is corroborated by the positive association found between the prevalence of symptoms of asthma, and paracetamol sales [12,23], and more recent work showing associations between symptoms of all three conditions and paracetamol use [23]. The analysis in European countries of *trans *fatty acids found a positive association, suggesting that man-made fats may be a factor in the prevalence of the three conditions [17].

### Tobacco

The picture which emerged for tobacco was mixed with no association observed with country tobacco consumption [11]. However there was generally a positive relationship between women smoking and the three conditions, yet an inverse association between men smoking and the three conditions. This analysis indicates that the well established individual level association between parental cigarette smoking and asthma does not account for the international differences in asthma prevalence.

### Tuberculosis

The inverse association found between asthma symptom prevalence and estimated TB incidence [13] and actual TB notifications rates [15] supports other evidence that exposure to *Mycobacterium tuberculosis *may reduce the risk of developing asthma. This may occur through induction of Th1 type immune responses. The implications of this relationship in the changing world of TB disease (the increase in AIDS and the concomitant increase in TB cases in Africa and the decrease of TB in other regions such as Latin America) need further study.

### Immunisation

Two levels of immunisation analyses were undertaken: country level, and centre level. The country level analyses showed no associations [7]. The more powerful centre-level analyses showed small inverse relationships between DTP and measles in the older age group only, with no associations with BCG. In view of earlier reports that immunisation might be a risk factor for asthma, this mainly null result is reassuring for population immunisation programmes, given their importance for child health.

### Antibiotics

The relationships between symptom prevalence and antibiotic exposure was not clear cut. A mixture of weak inverse and positive effects were found between symptom prevalences and total antibiotic sales and broad spectrum antibiotic sales [10]. This analysis suggested that even if there was a potential causal association of antibiotic use with asthma risk, it did not appear to explain the world wide differences between countries. The potential involvement, if any, of antibiotics may be in the alteration of induction of immunity rather than the exposure per se, and involve a combination of the effects of infection and microbial exposure too.

### Pollen

Exposure to allergenic pollen was assessed by exposures around the dates of early life [8] and did not appear to increase the risk of acquiring symptoms of respiratory allergy, and may even give some protection. Other studies have found that the symptom prevalence of hay fever and asthma tends to be lower in rural than in urban areas, and lowest among people living on farms [24-28], but this has not been consistently found outside Europe and USA, and was not studied in our analyses. The degree of consistency in the inverse associations suggests the possibility of a protective effect of pollen on allergy.

### Air pollution

A weak inverse relationship was demonstrated between city-level particulate air pollution (PM_10_) and symptoms of the three conditions, even after controlling for GNP which has a strong inverse association with air pollution [6]. Meta-analyses of data from countries with multiple centres found by contrast a consistent pattern of weak positive associations. These generally weak associations were in line with existing ecological evidence on the association between particulate air pollution and asthma. This finding is not incompatible with the extensive evidence from individual level studies that air pollution may aggravate existing asthma, since this may not have an important effect on prevalence. Short-term fluctuations in pollutant levels may have different effects from chronically high concentrations. Neither does it exclude a causal role for roadside exposure for which there is limited evidence. Higher self-reported exposure to truck traffic obtained as part of ISAAC Phase Three was associated with higher symptom prevalences [29].

### Climate

As climate affects whole populations, ecological studies are ideally suited to examine the relationship between prevalence of diseases and climatic conditions between populations. In the worldwide analyses few significant associations were seen [16]. However, in ISAAC studies in two large continents with quite marked climate differences - Latin America [30] and Africa [31] - no relationship was observed for asthma symptoms prevalence with respect to latitude, altitude, humid/dry climate or other geographical aspects, suggesting that meteorological and geographic factors, individually, would not be able to explain the wide variability in the prevalence of asthma, rhinitis and eczema in the world. As the world becomes more affected by climate change there may be some regions such as Western Europe where prevalence of disease is affected by potentially modifiable factors including humidity and temperature [16], but at a global level our ecological analyses showed little effect.

## Is the ecological approach sound?

The central ISAAC approach has been to study symptoms of disease *between *populations, which has naturally led to ecological analyses between symptom prevalences and potential environmental exposures. As Rose states, "the primary determinants of disease are mainly economic and social, and therefore its remedies must also be economic and social" [32], and this has been the thrust of the ISAAC approach. If the environment of populations is important in the occurrence of asthma, rhinoconjunctivitis and eczema, as the evidence suggests it is, analyses should be at the population environmental level. Thus ecological analyses are a most useful way to examine the effect of the social, economic and other aspects of the environment on health. As Marmot has argued, analyses of individual risk factors may be inappropriate if social environmental causes of illness are sought [33]. The ecological approach has been successful in suggesting hypotheses concerning possible causes of international patterns of cancer in the 1950s and 1960s, which were investigated in depth in further studies [34]. As some risk factors genuinely operate at the population level, either directly causing disease, or causing disease as effect modifiers or determinants of exposure to individual risk factors, the prediction of the health effects in an exposed population can be of primary importance [35].

Naturally these data must be interpreted with care. The potential problem with ecological studies is that the relationship between factors at an ecological level may not be the same as the relationship at an individual level. It cannot be assumed that exposure-disease associations in comparisons between populations necessarily represent associations at the individual level - the 'ecological fallacy' [36-38]. There may be several reasons for this, including population-level confounding (populations differ for many variables that cannot be included in the analysis), aggregation bias (grouping of disparate types of individuals), and misclassification bias. On the other hand, ecological studies are not susceptible to certain biases that can affect associations among individuals: for example, recall bias, when current disease influences memory of exposure, and reverse causality, if early symptoms lead to treatment (e.g. paracetamol) or behaviour change (e.g. smoking cessation by parents) that then appear to be adverse factors for the established disease. The ecological approach may be the only way to study the effect of whole population exposures, and the problems of interpreting individual-level data are perhaps less commonly appreciated.

Notwithstanding concerns about potential biases, the large number of countries, centres and children participating provides an opportunity to identify themes for further exploration relating to these three interrelated conditions. These analyses were undertaken to make ecological inferences about effects on population symptom prevalence rates, rather than biological inferences about effects on individual risks. In ISAAC Phase Three there was individual exposure ascertainment with a wider range of exposures providing the opportunity to conduct parallel individual and centre (population) level analyses, and further exploration of the ecological approach.

## Conclusion

ISAAC has explored environmental factors at a population level which may relate to the prevalence of asthma, rhinoconjunctivitis and eczema while recognising the limitations of the ecological approach in causal inference at the individual level. Some global associations which could be explored by further research were positive associations between the prevalence of symptoms of asthma, rhinoconjunctivitis and eczema in populations and GNP, *trans *fatty acids, paracetamol, and women smoking and inverse associations between food of plant origin, pollen, immunisations, tuberculosis notifications, air pollution, and men smoking, and some showed mixed effects (antibiotics and climatic factors). While the global ecological approach has advantages, it may miss factors of importance within regions, and involving combinations of variables within or between regions. ISAAC Phase Three has extended research in the areas identified in this review using an environmental questionnaire which enables some of these hypotheses to be explored further with individual level as well as ecological analyses. Further studies could include randomised controlled trials of putative risk factors such as paracetamol exposure. Since these ecological studies took place, globalization has introduced further large environmental changes. Rapid environmental and lifestyle changes within whole societies offers potential opportunities for demonstrating the importance of community-wide determinants of ill-health, and provides a rationale for monitoring time trends in asthma and allergic diseases in diverse populations, as in Phase One centres that also participated in ISAAC Phase Three.

## Abbreviations

AIDS: Acquired Immune Deficiency Syndrome; BCG: Bacille Calmette-Guérin vaccination; DTP: Diphtheria, tetanus and pertussis vaccination; ISAAC: The International Study of Asthma and Allergies in Childhood; PM_10_: Particulate matter with aerodynamic diameter < 10 μm; TB: Tuberculosis; Th1: T helper cell 1; WHO: World Health Organization.

## Competing interests

The authors are co-authors on the ISAAC publications referred to in this article. They declare no competing interests.

## Authors' contributions

IA drafted the manuscript. The named authors on the title page made a large contribution to the manuscript writing. All authors contributed data to the studies, and read, commented and approved the final manuscript.

## Authors' information

Professor Innes Asher is Head of the Department of Paediatrics: Child and Youth Health at the University of Auckland and has been Chair of the ISAAC Steering Committee since its inception in 1991. Alistair Stewart is a senior Biostatistician at the University of Auckland and is a member of the ISAAC Steering Committee. Professor Javier Mallol is Head of the Department of Paediatric Respiratory Medicine at Hospital CRS El Pino, University of Santiago de Chile (USACH). Professor Mallol is the ISAAC Regional Coordinator for Latin America and is a member of the ISAAC Steering Committee. Professor Stephen Montefort is a Consultant Respiratory Physician and Professor of Medicine in the Department of Medicine, University of Malta. Professor Montefort is the ISAAC Regional Coordinator for the Eastern Mediterranean and is a member of the ISAAC Steering Committee. Professor Christopher Lai is an honorary clinical professor in the Department of Medicine and Therapeutics, the Chinese University of Hong Kong. Professor Lai is the ISAAC Regional Coordinator for the Asia-Pacific region and is a member of the ISAAC Steering Committee. Professor Nadia Aït-Khaled is Head of Asthma Division of the International Union Against Tuberculosis and Lung Disease (The Union). Professor Aït-Khaled is the ISAAC Regional Coordinator for French-speaking Africa and is a member of the ISAAC Steering Committee. Dr Joseph Odhiambo is based at the Centre for Respiratory Diseases Research, Kenya Medical Research Institute (KEMRI), Nairobi. Dr Odhiambo is the ISAAC Regional Coordinator for English-speaking Africa and is a member of the ISAAC Steering Committee.
